# Implication of FDG-PET/CT in patients with potentially operable colorectal lung metastases

**DOI:** 10.1515/iss-2021-0029

**Published:** 2021-12-24

**Authors:** Anton Uporov, Samantha Taber, Lope Estèvez Schwarz, Joern Groene, Lothar R. Pilz, Gregor Foerster, Roland Bittner, Joachim Pfannschmidt

**Affiliations:** Department of Thoracic Surgery, Heckeshorn Lung Clinic – HELIOS Klinikum Emil von Behring, Berlin, Germany; Department of Surgery, St. Joseph-Hospital, Berlin, Germany; Medical Faculty Mannheim, Heidelberg University, Mannheim, Germany; Department of Radiology and Nuclear Medicine, HELIOS Klinikum Emil von Behring, Berlin, Germany

**Keywords:** colorectal cancer, lung metastasis, PET/CT, staging, thoracic surgery

## Abstract

**Objectives:**

This prospective study assessed the role of F-18-FDG-PET/CT in clinical staging for patients with colorectal cancer planned for pulmonary metastasectomy by thoracotomy or video-assisted surgery.

**Patients and methods:**

In addition to conventional imaging, we performed 86 F-18-FDG-PET/CT studies in 76 patients with potentially resectable metastatic colorectal lung metastases. We then investigated the effect that PET/CT had on further clinical management. Based on the results from the 47 thoracotomies performed, we compared the number of pulmonary metastases discovered after histologic examination with the number predicted by the conventional computed tomography (CT) as an independent part of the F-18-FDG-PET/CT examination and by the F-18-FDG-PET component.

**Results:**

F-18-FDG-PET/CT led to changes in treatment regime and diagnostic planning in many patients. In five patients PET/CT revealed previously undetected local recurrence of the primary colorectal cancer, in four patients hepatic metastases, in three patients bone metastases, in two patients soft-tissue metastases, and in three patients histologically preoperatively proven N2 or N3 station lymph node involvement. These all constituted exclusion criteria, and consequently the previously planned pulmonary metastasectomy was not performed. The sensitivity and positive predictive value (PPV) for detection of pulmonary metastases were 84.2% and 36.4% for CT and 75.0% and 61.6% for F-18-FDG-PET study. The calculated sensitivity, specificity, PPV, and NPV of F-18-FDG-PET/CT for detecting thoracic lymph node involvement were 85.7%, 93.0%, 66.7%, and 97.5%, respectively. Furthermore, we found that F-18-FDG-PET/CT may predict thoracic lymph node involvement based on the SUV of pulmonary nodules.

**Conclusions:**

F-18-FDG-PET/CT has a clear role in the diagnostic workup for pulmonary metastatic colorectal cancer and may save patients from futile surgery. It cannot, however, be relied on to detect all possible pulmonary and nodal metastases, which surgeons must always consider when making treatment decisions.

## Introduction

Surgical removal of pulmonary metastases from colorectal cancer remains the mainstay of treatment for a highly selected subset of patients with isolated pulmonary metastases. Five-year survival rates following pulmonary metastasectomy have been reported at around 50% with a low operative mortality of <2% [1]. Identifying which patients will truly benefit from pulmonary metastasectomy, however, is paramount. Computer tomography (CT) is the most accepted method for the detection of such pulmonary metastases [2, 3], and the main reason for exclusion from surgical treatment (with rare exceptions) is presence of extrathoracic disease, including primary site relapse. Although conventional CT alone is often employed, preoperative F-18 fluorodeoxyglucose (FDG) PET/CT can play an important role in modifying the clinical management of patients with lung metastases. Not only can it detect occult metastases in other organs, primary site recurrence, and nodal spread at the hilar or mediastinal level, but it can also help differentiate between malignant and benign pulmonary nodules. The aim of this study was to evaluate the clinical value of F-18-FDG-PET/CT in the preoperative staging of lung metastases from colorectal cancers.

## Methods

This prospective, nonrandomized study ran from 2014 to 2019 and included all patients referred to the thoracic surgery multidisciplinary team at Lung Clinic Heckeshorn as candidates for pulmonary metastasectomy of lesions from primary colorectal cancers. Recommendation for evaluation was based on previous conventional staging procedures (thoracic and abdominal CT scans or abdominal ultrasound; also, colonoscopy in selected patients). All patients eligible for pulmonary metastasectomy and for F-18-FDG-PET/CT examination were included in the study. A total of 76 patients with 86 PET/CT scans were included. There were 44 men and 32 women with a mean age of 67 years (28–83 years).

F-18-FDG-PET/CT images were evaluated by thoracic (R.B.) and nuclear radiologists (G.F.). All patients were informed of the objectives of the study and gave their informed consent. The study protocol was approved by the ethics committee of the Berlin Aerztekammer (Eth-21/14).

We recorded the number of nodules detected in CT and F-18-FDG-PET separately. Lymph nodes were considered PET-positive if they demonstrated an uptake higher than the mediastinal blood pool on F-18-FDG-PET. Since complete palpation was deemed necessary for determining the correct number of pulmonary nodules, only patients with thoracotomies were included in this analysis (no resections per VATS). Pathology reports concerning the number of nodules and affected lymph nodes were then obtained and used as the reference for determining the sensitivity and positive predictive value of CT and F-18-FDG-PET. Additional, preoperative patient assessment, surgical approach, and surgical technique were performed as described in Table 1.

**Table 1:** Patient characteristics at PET/CT.

Demographics	All patients
Age, years	67.6 ± 10.3
Sex	
Female	36 (42.0%)
Male	50 (58.0%)
Site of primary colorectal cancer	
Colon	29 (34%)
Rectal	57 (66%)
Tumor differentiation colorectal cancer primary	
Well differentiated	3 (3%)
Moderately differentiated	53 (62%)
Poorly differentiated	11 (13%)
Unknown	19 (22%)
pT colorectal cancer primary	
T0	4 (5%)
T1	4 (5%)
T2	12 (14%)
T3	55 (64%)
T4	7 (8%)
Unknown	4 (5%)
pN colorectal primary	
Node+	52 (60%)
Node−	28 (33%)
Unknown	6 (7%)
Carcinoembryonic antigen	
0–5 ng/mL	52 (61%)
>5 ng/mL	21 (24%)
Unknown	13 (15%)
Chemotherapy colorectal cancer primary	
Preoperative	43 (50%)
Postoperative	39 (45%)
None	4 (5%)
Thoracic lymph node involvement by PET	
Node+	18 (21%)
Node−	68 (79%)
Pulmonary nodes detected by FDG-PET	141
Pulmonary nodes detected by CT	234
Pulmonary nodes resected (69 procedures)	147
Pulmonary metastases CRC	97
Different histology	50
Primary lung cancer	8
Treatment pulmonary nodules after PET/CT	
Surgical resection	69 (81%)
No-local treatment (no surgery)	17 (19%)
Number of pulmonary lesions surgical resected per procedure	
1	38
>1	31
Surgical approach	
Unilateral	56
Bilateral/staged	13
Thoracic lymph node dissection	
No LND	19 (22%)
Systematic LND	40 (47%)
Lymph node sampling	10 (12%)
No surgery	17 (19%)
Thoracic metastatic lymph node involvement (histopathology)	
Node positive	7 (9%)
Node negative	33 (38%)
Unknown (no systematic LND or no surgery)	46 (53%)

### Surgical criteria

Patients are considered viable candidates for pulmonary metastasectomy if the lesions are technically resectable, the patients themselves are fit enough for an intrathoracic operation, and there is no presence of extrathoracic disease, including local recurrence of the colorectal malignancy (the exception here is hepatic lesions, which can also be resected along with pulmonary metastases in curative intention).

### Main measures

The primary target of this study was to determine the role of F-18-FDG-PET/CT in detecting pulmonary metastases, thoracic lymph node involvement, and possible extrathoracic disease in patients with primary colorectal cancers. Our primary points of interest were sensitivity, specificity, positive-predictive value (PPV), and negative-predictive value (NPV) for both F-18-FDG-PET- and for conventional CT-analysis.

### Statistical analysis

All statistical analyses were performed using the statistical software SAS 9.4, SAS Institute Inc., Cary, NC, USA. For dichotomous and ordinal variables we report absolute and relative number along with 95%-confidence intervals (95%-CI), based on the “Wald-Method” [4]. For continuous variables we report absolute number, mean ± standard deviation, median, range, skewness, and kurtosis. Further, we calculated sensitivity, specificity, PPV and NPV for both F-18-FDG-PET- and conventional CT as a means of detecting nodal metastases (mediastinal or hilar). For sub-group analysis the exact Fisher-test was used for dichotomous and ordinal data, and the χ^2^-test was used for continuous data. Sensitivity and specificity for CT and F-18-FDG-PET were calculated using the histologically determined number of metastatic lesions as the reference. We also performed receiver operating curve analysis (ROC-analysis) to investigate the positive occurrence of malignant lymph nodes based on SUV-data for pulmonary nodules. Here, we also calculated area under curve (AUC), as well as numbers of concordance, discordance, and bindings. Potential influencing factors were analyzed with logistic regression in down step selection method. Maximum likelihood estimates were used, as well as the Hosmer–Lemeshow-test to judge the stability of the regression model, where higher p-values indicate a more reliable model. Odds ratio estimates including Wald-95%-CIs were used to demonstrate possible influencing factors. The level of statistical significance was set to α=0.05.

## Results

During the study period, 86 F-18-FDG-PET/CT studies were performed. Seven patients had two F-18-FDG-PET/CT scans, and one patient had four F-18-FDG-PET/CT studies due to recurrent pulmonary disease. 33 (38%) F-18-FDG-PET/CT studies were initiated for synchronous pulmonary metastases and 53 (62%) for metachronous disease. 22 F-18-FDG-PET/CT-examinations prompted modifications to treatment regime or led to additional diagnostics. In 8 patients F-18-FDG-PET/CT detected further pulmonary or lymphatic progression, and in 14 patients new extrathoracic lesions were suspected. Five patients had local recurrence of the primary colorectal cancer, four had hepatic metastases, three had bone metastases, and two had soft-tissue metastases. FDG-avid lymph nodes were detected in five N1 stations, five N2 stations and six N3 stations. In one patient with N3 F-18-FDG -avid lymph nodes and in two patients with N2 18-F-FDG -avid lymph nodes preoperative histological analysis confirmed metastatic lymphatic involvement, and these patients were excluded from initially planned surgical metastasectomy. Thus, F-18-FDG-PET/CT prevented 17 (19%) patients from receiving surgery, which they did not stand to benefit from.

Conventional CT (as part of the F-18-FDG-PET/CT scan) detected a greater number of pulmonary nodules (236 nodules) than F-18-FDG-PET (141 nodules) (p=0.0001). In 47 examinations conventional CT and F-18-FDG-PET revealed the same number of pulmonary lesions. 37 (43%) CT and 52 (60%) F-18-FDG-PET studies revealed solitary pulmonary lesions. 75 patients (87.2%) had F-18-FDG-avid pulmonary lesions with SUVmax of >2.5. The mean SUVmax in all patients with 18F-FDG avid lesions was 5.7 ± 4.2.

In 47 patients F-18-FDG-PET/CT led to open surgical procedures being performed. Of all nodules resected (n=207), 128 were pulmonary metastases from colorectal cancers, while 79 nodules turned out to have other histologies (eight were primary non-small cell lung cancers). In 21 cases CT predicted the correct number of pulmonary metastases (with no additional lesions of other histology identified), and in 43 cases F-18-FDG-PET predicted the number correctly.

**Figure 1:**
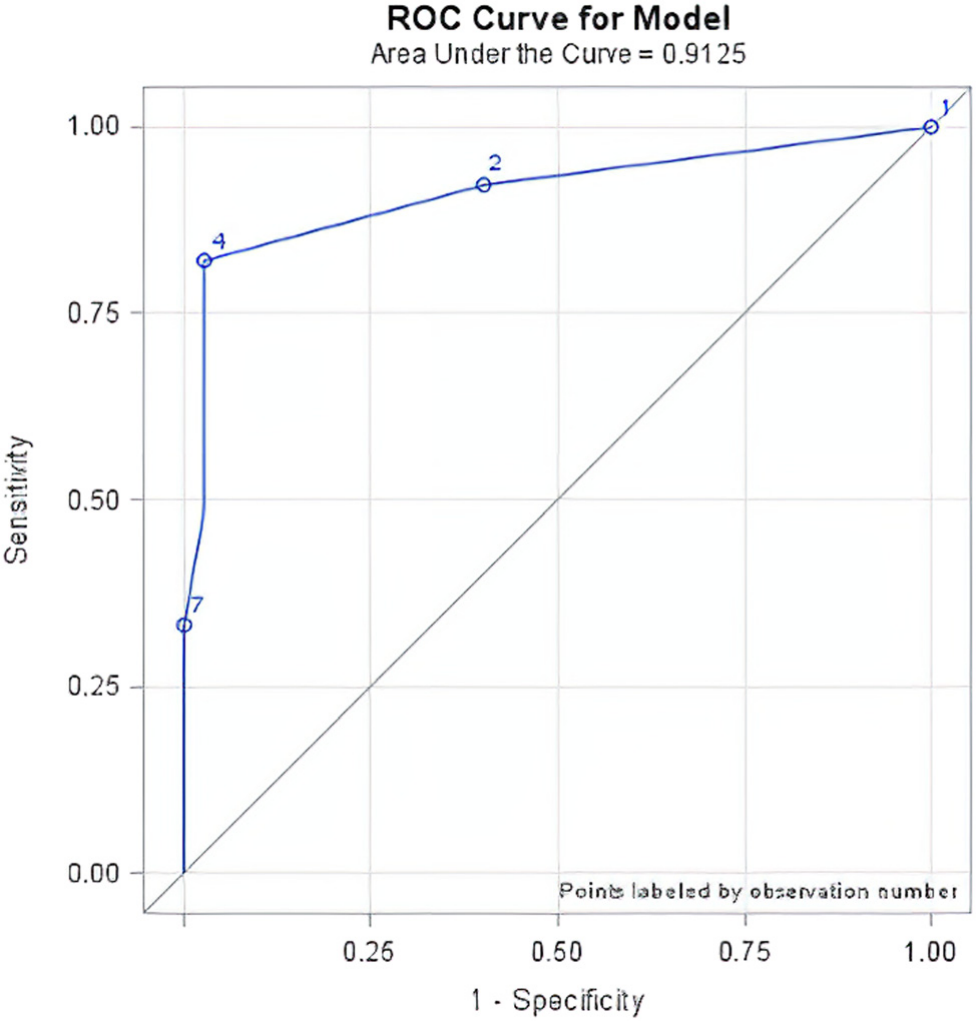
ROC curve of minimal SUV mean in pulmonary lesions and the detection mediastinal or hilar lymph node involvement (area under the curve (AUC) 0.9125).

**Figure 2:**
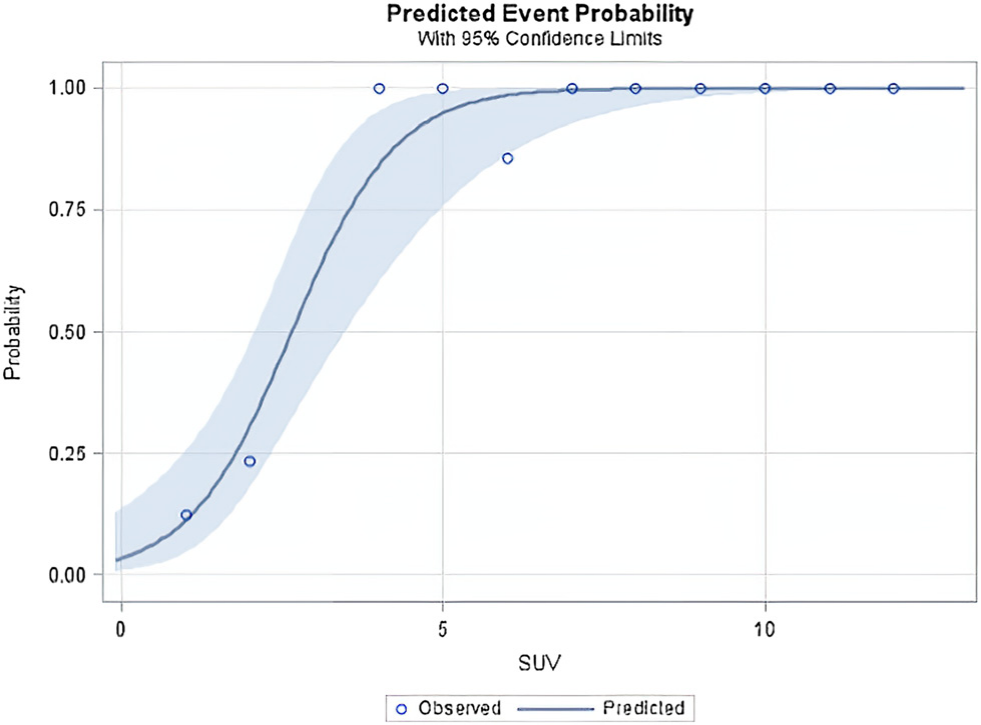
Predicted event probability for lymph nodes involvement in correlation with the SUVmean of pulmonary lesions (blue ribbon represents 95% confidence intervals).

Using the number of histologically verified number of metastases as the reference we found that CT had a sensitivity of 84.2% and a positive predictive value of 36.4%. For F-18-FDG-PET the sensitivity was 75.0% and the positive predictive value (PPV) 61.6%-Specificity and negative predictive value (NPV) could not be determined, since false-negative cases were not registered. Of the 50 patients treated with pulmonary metastasectomy and systematic lymph node dissection or lymph node sampling, 10 patients had F-18-FDG -avid hilar or mediastinal lymph nodes; in six cases metastatic involvement from the primary colorectal cancer was confirmed histologically. In one case F-18-FDG-PET failed to detect an N1 lymph node metastasis, which was confirmed by histological examination. Again, using histological results as the reference for detecting nodal metastases, F-18-FDG-PET/CT had a sensitivity of 85.7%, a specificity of 93.0%, a PPV of 66.7%, and a NPV of 97.5% (Table 2). We performed a ROC analysis to calculate the likelihood that a PET-avid lung lesion would predict metastatic lymph node involvement (Figure 1). Further, we found that F-18-FDG-PET/CT as a means of predicting pulmonary lymph node involvement based on the SUV of pulmonary nodules had a sensitivity of 97.4%, a specificity of 87,.2% (Figure 2), a positive predictive value of 86.3%, and a negative predictive value of 97.6%.

**Table 2:** Diagnostic performance: Sensitivity, specificity, positive predictive value (PPV), and negative predictive value of F-18-FDG-PET.

F-18-FDG-PET	Number of pulmonary metastases	Lymph node metastases
Sensitivity	75.0%	87.5%
Specificity	N.A.	87.0%
PPV	61,6%	70%
NPV	N.A.	97.6%

Although the parameters age, primary cancer grade, serum CEA levels, and SUVmax were included in the logistic regression model and subgroup analysis, none had a significant effect on clinical management.

## Discussion

Metastasectomy of pulmonary lesions in selected patients with colorectal cancer has an established role in prolonging survival. Although the CT-scan is used for detecting synchronous or metachronous pulmonary metastases [5] it is ill-equipped for distinguishing between benign and malignant pulmonary nodules or for revealing extrapulmonary metastatic disease. The objective of this study was to evaluate the value of F-18-FDG-PET/CT in the preoperative staging of colorectal cancer patients considered for curative pulmonary metastasectomy. Currently, F-18-FDG-PET/CT is used to determine the metabolic activity of suspect lung nodules and to determine whether lymph nodes or extrathoracic regions are affected. Although more and more thoracic surgeons are using F-18-FDG PET/CT as a means of selecting colorectal cancer patients for metastatectomy [6], its precision has only rarely been investigated [7]. Some studies on the role of F-18-FDG-PET/CT in the selection of patients with potentially resectable metastatic hepatic disease have demonstrated that F-18-FDG PET/CT improves the accuracy of preoperative staging, thus avoiding unnecessary surgery in patients, who will not benefit from it. Selzner et al. found [8] that in patients with potentially resectable liver metastases F-18-FDG-PET/CT had a sensitivity of 89%, compared to 64% for CT alone to detect extrahepatic disease, and was thus estimated as able to prevent non-useful surgery in 21% of patients. Desai DC [9] and colleagues reported that F-18-FDG-PET/CT was 72% more effective at discovering more extensive disease in patients, previously thought to have isolated liver involvement, here too avoiding futile surgery. Interestingly, Lubezky et al. [10] in contrast found that contrast enhanced CT was more sensitive than F-18-FDG-PET/CT at detecting hepatic metastases from colorectal cancers, but only after neoadjuvant therapy. In our study, no neoadjuvant chemotherapy in a multimodality approach before evaluation for pulmonary metastasectomy by F-18-FDG-PET/CT has been applied. Thus, we could not investigate the role of chemotherapy and the sensitivity of F-18-FDG-PET under these specific circumstances. Nevertheless, the expectation that chemotherapy influences the metabolic activity of lung metastases and thus reduces the sensitivity of F-18-FDG-PET seems to be justified.

Currently, international guidelines recommend F-18-FDG-PET/CT as a means of ruling out recurrence of colorectal cancers in patients under consideration for curative pulmonary metastasectomy [11].

Regarding pulmonary lesions the situation is more complicated. Yu et al. [12], demonstrated that F-18-FDG-PET/CT was useful in identifying pulmonary nodules. Although preoperative biopsies from suspicious pulmonary nodules can be useful, they are not always possible. In these situations, F-18-FDG-PET/CT scan aid in decision making, but it is not without limitations. While the majority of PET-avid lesions in our study were histologically confirmed as metastases, in eight cases the lesion in question turned out to be primary non-small cell lung cancer, and in six cases lesions turned out to be benign.

Our results showed that in 57.6% of surgically resected patients F-18-FDG-PET failed to determine the exact number of metastases. The high frequency of false negatives is likely due to the smallness of many metastatic lesions. Not only are they often below the resolution of conventional PET/CT scanners, but this problem is also exacerbated by a non-breath hold/non-gated imaging technique. Nevertheless, F-18-FDG-PET was still more frequently correct in estimating the number of metastatic lesions than conventional CT. This highlights its role as a complementary examination, but one that cannot be used to make treatment decisions alone.

Although patients included in our study had already received full preoperative workups, F-18-FDG-PET/CT proved beneficial in 17 patients (19%) by identifying previously undiagnosed intra- or extra-pulmonary metastatic disease and preventing futile surgery.

The other compelling indication for F-18-FDG-PET/CT in colorectal cancer patients being considered for metastasectomy is to detect nodal involvement. Fong et al. [13], however, found that preoperative F-18-FDG-PET/CT for planned hepatic metastasectomy was not superior to CT in detecting lymph node metastases. Nevertheless, thoracic lymph node involvement has such a significant negative effect on overall survival in patients with pulmonary metastasectomies [14, 15] that we decided to investigate whether F-18-FDG-PET/CT could accurately predict intrathoracic nodal metastases. Concerning lymph node evaluation, 7 (17.5%) of 40 patients in our investigation had lymph node metastases, diagnosed on the basis of systematic lymph node dissection: 1 (2.5%) isolated N1 station, 4 (10%) N1 + N2 stations, and 2 (5%) isolated N2 stations. Bölükbas and associates [16] reported on 165 patients and Welter et al. [17] on 175 patients with pulmonary metastases of colorectal origin and found 22.4% and 16.7% lymph node involvement after systematic lymph node dissection. Cases of pulmonary or hilar lymph node involvement (which represents the drainage of the lung) are often interpreted as secondary metastases from primary pulmonary metastases. The competing hypothesis, that mediastinal lymph node involvement results from abdominal spread (namely, celiac, portal lymph nodes) [18] is not supported by our study, as our patients with N2 involvement did not display any F-18-FDG -avidity in the abdominal lymph nodes. We found that F-18-FDG-PET had high rates of sensitivity (85.7%) and specificity (93.0%) for detecting mediastinal or hilar lymph node involvement – much higher than reported findings on abdominal lymph node metastases. A meta-analysis by Dahmarde et al. [19], reports 89% sensitivity and 69% specificity for F-18-FDG-PET/CT in detecting abdominal lymph node metastases. The authors, however, report a high degree of heterogeneity in the 13 studies reviewed and suggest that this may have been a potential source of bias. They also speculate that increased FDG uptake may have been due to abdominal inflammation, recent chemoradiotherapy, and recent surgery and suggest prospectively designed studies that use a SUV max cut-off of ≤2.5 for lymph node involvement. In our study the mean SUV max of analyzed lymph nodes was 6.6 (SD 2.4). Recently, in a large retrospective study, 344 patients with colorectal lung metastases were evaluated for potential intrathoracic lymph node involvement [20]. Here, F-18-FDG-PET/CT had a sensitivity of 34%, specificity of 98%, positive predictive value of 88%, and negative predictive value of 78%. Considering the limited sensitivity of F-18-FDG-PET/CT, we must conclude that PET-negative lymph nodes do not justify forgoing systemic lymph node dissection. Interestingly, however, we found that the SUV of pulmonary metastasis could help predict occult lymph node metastasis. This has been described for primary lung cancer and other cancer entities but has not previously been addressed for pulmonary metastatic disease and thoracic lymph nodes. Thus, for patients with morphologically normal lymph nodes by CT or PET/CT further preoperative lymph node staging by bronchoscopy or mediastinoscopy may be important.

The main limitation of our study is its highly selected patient population. The patients included and evaluated for pulmonary metastasectomy were drawn from a regular follow-up program. In this regard, sensitivity, specificity, and positive predicted value of F-18-FDG-PET may be overestimated. Moreover, the small number of surgically treated patients may affect the significance of the observed parameters. Finally, it was not possible to consider the biological risk factors for colorectal cancer recurrence after pulmonary metastasectomy, as proposed recently by Corsini et al. [21].

In conclusion, F-18-FDG-PET/CT has a clear role in the diagnostic work-up of patients with suspected pulmonary metastatic colorectal cancers. Most importantly, it may save some patients from futile surgery, as CT alone can lead to an understaging in the presence of extrapulmonary disease. However, the present study also gives a clear indication that F-18-FDG-PET/CT have limitations and cannot detect all possible lung metastases. Moreover, their ability to detect metastatic lymph nodes is limited. With these considerations in mind clinicians should combine F-18-FDG-PET/CT with other clinical methods like bronchoscopy or mediastinoscopy to arrive at the best possible management plan for each individual patient.

## Supplementary Material

Supplementary Material
