# Supplementary material for: Implication of FDG-PET/CT in patients with potentially operable colorectal lung metastases
Source: Innov Surg Sci. 2021 Dec 24;6(3):89–95. doi: 10.1515/iss-2021-0029 (PMC8826161; doi:10.1515/iss-2021-0029)
Supplement: Supplementary file 1 — Supplementary Material [file j_iss-2021-0029_suppl.pdf]

Reviewer Assessment

J. Pfannschmidt et al.: Implication of FDG-PET/CT in patients with potentially operable colorectal lung metastases

Reviewers’ Comments to Original Submission

Reviewer 1: anonymous

Date received: 16-Aug-2021
Reviewer recommendation: **Return to author for minor modifications**
Reviewer overall scoring: **Medium**

Assessment Form scores: 5 = High/Yes; 3 = Medium/Adequate; 1 = Low

Is the subject area appropriate for the journal		4			
Does the title clearly reflect the paper's content?	5				
Does the abstract clearly reflect the paper's content	5				
Do the keywords clearly reflect the paper's content?	5				
Does the introduction present the problem clearly?		4			
Are the results/ conclusions justified?	5				
How comprehensive and up-to-date is the subject matter presented?		4			
How adequate is the data presentation?	5				
Are units and terminology used correctly?	5				
Is the number of cases adequate?			3		
Are the experimental methods/ clinical studies adequate?			3		
Is the length appropriate in relation to the content?					1
Does the reader get new insights from the article?				2	
Please rate the practical significance.			3		
Please rate the accuracy of methods.		4			
Please rate the statistical evaluation and quality control.		4			
Please rate the appropriateness of the figures and tables.	5				
Please rate the appropriateness of the references.		4			
Please evaluate the writing style and use of language.			3		
Please judge the overall scientific quality of the manuscript.			3		
Are the methods used worthy of reproduction in greater deal?	No				
Would you be willing to review a revision of this manuscript?	Yes				

Comments to author: The results are clearly presented and they reflect the experience of most physicians involved in pulmonary metastasectomy.

However, the length of the paper exceeds by far the one required for conveying the message. A substantial shortening - especially of the discussion - is advisable.

Reviewer 2: Niesen, Andreas

Date received: 16-Aug-2021
Reviewer recommendation: **Return to author for minor modifications**
Reviewer overall scoring: **High**

Assessment Form scores: 5 = High/Yes; 3 = Medium/Adequate; 1 = Low

Is the subject area appropriate for the journal	5				
Does the title clearly reflect the paper's content?	5				
Does the abstract clearly reflect the paper's content	5				
Do the keywords clearly reflect the paper's content?	5				
Does the introduction present the problem clearly?	5				
Are the results/ conclusions justified?	5				
How comprehensive and up-to-date is the subject matter presented?		4			
How adequate is the data presentation?		4			
Are units and terminology used correctly?		4			
Is the number of cases adequate?			3		
Are the experimental methods/ clinical studies adequate?	5				
Is the length appropriate in relation to the content?	5				
Does the reader get new insights from the article?		4			
Please rate the practical significance.		4			
Please rate the accuracy of methods.	5				
Please rate the statistical evaluation and quality control.	5				
Please rate the appropriateness of the figures and tables.		4			
Please rate the appropriateness of the references.				2	
Please evaluate the writing style and use of language.			3		
Please judge the overall scientific quality of the manuscript.			3		
Are the methods used worthy of reproduction in greater deal?	Yes				
Would you be willing to review a revision of this manuscript?	Yes				

Comments to author: -the literature is partially quite old (e.g. 1999)
-the typicall limitations of pet as well as of ct are well known and should be discussed so the results can be classified better (e.g. lit 13 on page 14).

Authors' Response to Reviewer Comments

Date received: 20-Sep-2021

Response to reviewer 1

Reviewer 1 asked to shorten the length of the manuscript, especially of the discussion:
According to the suggestion of Reviewer 1, we streamlined the content of the manuscript by deleting the following sentences:
# Mediastinal lymph node involvement has also been suggested as another important ominous prognostic factor in otherwise resectable colorectal lung metastases [4][5], underscoring the need for accurate staging of both extrathoracic sites and mediastinal nodes. Only then is it possible to select the patients, who are likely to benefit from pulmonary metastasectomy. (Page5)

# Sacks and colleagues [15], too, highlighted the role of F-18-FDG-PET/CT in ruling out metastatic spread from colorectal cancers. (Page13)

# Bae and colleagues also [17] found that F-18-FDG-PET/CT had a low sensitivity for detecting regional lymph node metastases. They speculated that this may be due to the partial volume effect that spills radioactivity into the background of small lesions and makes the actual standardized uptake value (SUV>2.5) insignificant. (Page15)

# In earlier studies Pfannschmidt et al. found thoracic lymph node metastases in 19.2% of 167 patients with pulmonary metastatic disease from colorectal carcinomas [20]. (Page15)

# Nevertheless, the specific intrathoracic location affected may be significant. Pfannschmidt and colleagues [23] found that in patients with colorectal primaries, median survival after pulmonary metastasectomy, when only hilar lymph nodes were affected, was 21 months – versus 15 months when mediastinal lymph nodes were involved. (Page16)

#In a small subgroup analysis of 7 patients with mediastinal lymph node metastases of different histologies, Pastorino et al. [25] found that F-18-FDG-PET had a sensitivity of 100%, accuracy of 96%, and negative predictive value of 100%. (Page17)
# Missing occult lymph nodes could mean missing an opportunity to interrupt the cascade of metastatic cells (Page16)

Response to reviewer 2

Reviewer 2 criticized, that the literature is partially quite old:
According to the suggestion by Reviewer 2, we try to update the literature:

# We replaced Fong (Fong Y, Saldinger PF, Akhurst T, MacApinlac H, Yeung H, Finn RD, et al. Utility of 18F-FDG positron emission tomography scanning on selection of patients for resection of hepatic colorectal metastases. Am J Surg 1999;178:282–7) and Truant (Truant S, Huglo D, Hebbar M, Ernst O, Steinling M, Pruvot FR. Prospective evaluation of the impact of [18F]fluoro-2-deoxy-D- glucose positron emission tomography of resectable colorectal liver metastases. Br J Surg 2005;92:362–9) with the more recent literature from Selzner et al. (Selzner M, Hany TF, Wildbrett P, Mccormack L, Kadry Z, Clavien P-A. Does the Novel PET/CT Imaging Modality Impact on the Treatment of Patients With Metastatic Colorectal Cancer of the Liver? Ann Surg 2004;240:1027-1036)

# We replaced Saito (Saito Y, Omiya H, Kohno K, Kobayashi T, Itoi K, Teramachi M, et al. Pulmonary metastasectomy for 165 patients with colorectal carcinoma: A prognostic assessment. J Thorac Cardiovasc Surg 2002;124:1007–13.) by Bölükbas (Bölükbas S, Sponholz S, Kudelin N, Eberlein M, Schirren J. Risk factors for lymph node metastases and prognosticators of survival in patients undergoing pulmonary metastasectomy for colorectal cancer. Ann Thorac Surg 2014;97:1926–32.)

# We replaced Vetto (Vetto JT, Cohen AM. Isolated spread of hepatic metastatic disease to a mediastinal lymph node - Report of a case and review of pertinent anatomy and literature. Dis Colon Rectum 1991;34:1128–30) and Kura (Kura H, Sato N,

Uchiyama A, Nakafusa Y, Mibu R, Yoshida K, et al. Mediastinal lymph node metastasis of colon cancer: Report of a case. Surg Today 1999;29:375–7) with the more recent publication by Toda (Toda K, Kawada K, Sakai Y, Izumi H. Metachronous mediastinal lymph node metastasis from ascending colon cancer: A case report and literature review. Int J Surg Case Rep 2017;41:336–9).

We, in addition, discussed literature 13 on page 14 accordingly:

„Interestingly, Lubezky et al. [13] in contrast found that contrast enhanced CT was more sensitive than F-18-FDG-PET/CT at detecting hepatic metastases from colorectal cancers, but only after neoadjuvant therapy. In our study, no neoadjuvant chemotherapy in a multimodality approach before evaluation for pulmonary metastasectomy by F-18-FDG-PET/CT has been applied. Thus, we could not investigate the role of chemotherapy and the sensitivity of F-18-FDG-PET under these specific circumstances. Nevertheless, the expectation that chemotherapy influences the metabolic activity of lung metastases and thus reduces the sensitivity of F-18-FDG-PET seems to be justified. "

Reviewers' Comments to Revised Submission

Reviewer 1: anonymous

Date received: 05-Oct-2021
Reviewer recommendation: **Accept in present form**
Reviewer overall scoring: **High**

Assessment Form scores: 5 = High/Yes; 3 = Medium/Adequate; 1 = Low

Is the subject area appropriate for the journal	5				
Does the title clearly reflect the paper's content?	5				
Does the abstract clearly reflect the paper's content	5				
Do the keywords clearly reflect the paper's content?	5				
Does the introduction present the problem clearly?		4			
Are the results/ conclusions justified?		4			
How comprehensive and up-to-date is the subject matter presented?		4			
How adequate is the data presentation?		4			
Are units and terminology used correctly?		4			
Is the number of cases adequate?			3		
Are the experimental methods/ clinical studies adequate?		4			
Is the length appropriate in relation to the content?			3		
Does the reader get new insights from the article?		4			
Please rate the practical significance.		4			
Please rate the accuracy of methods.		4			
Please rate the statistical evaluation and quality control.		4			
Please rate the appropriateness of the figures and tables.		4			
Please rate the appropriateness of the references.		4			
Please evaluate the writing style and use of language.	5				
Please judge the overall scientific quality of the manuscript.			3		

Are the methods used worthy of reproduction in greater deal?	Yes
Would you be willing to review a revision of this manuscript?	Yes

Comments to author: The authors made changes in the manuscript according to the reviewer´s suggestions, I do not have further comments.

Reviewer 2: Niesen, Andreas

Date received: 05-Oct-2020
Reviewer recommendation: **Accept in present form**
Reviewer overall scoring: **High**

Assessment Form scores: 5 = High/Yes; 3 = Medium/Adequate; 1 = Low

Is the subject area appropriate for the journal	5				
Does the title clearly reflect the paper's content?	5				
Does the abstract clearly reflect the paper's content	5				
Do the keywords clearly reflect the paper's content?	5				
Does the introduction present the problem clearly?	5				
Are the results/ conclusions justified?	5				
How comprehensive and up-to-date is the subject matter presented?	5				
How adequate is the data presentation?		4			
Are units and terminology used correctly?		4			
Is the number of cases adequate?			3		
Are the experimental methods/ clinical studies adequate?	5				
Is the length appropriate in relation to the content?	5				
Does the reader get new insights from the article?		4			
Please rate the practical significance.		4			
Please rate the accuracy of methods.	5				
Please rate the statistical evaluation and quality control.	5				
Please rate the appropriateness of the figures and tables.		4			
Please rate the appropriateness of the references.		4			
Please evaluate the writing style and use of language.			3		
Please judge the overall scientific quality of the manuscript.		4			
Are the methods used worthy of reproduction in greater deal?	Yes				
Would you be willing to review a revision of this manuscript?	Yes				

Comments to author: thanks for changes
